# Squamous Cell Carcinoma of the Esophagus in an Adolescent: A Case Report

**DOI:** 10.7759/cureus.68660

**Published:** 2024-09-04

**Authors:** Aayush Lakkanna, Vivek Gopalam, Guguloth Rajender, Souvik Manna

**Affiliations:** 1 General Surgery, Postgraduate Institute of Medical Education and Research (PGIMER), Chandigarh, IND; 2 Neurosurgery, Postgraduate Institute of Medical Education and Research (PGIMER), Chandigarh, IND; 3 Community Medicine, Employee's State Insurance Corporation (ESIC) Medical College and Hospital, Alwar, IND

**Keywords:** gi cancer, cancer in adolescent, gi oncology, nacrt, squamous cell carcinoma esophagus

## Abstract

Esophageal cancer is more common with increasing age and rarely seen below the age of 45. The current case report is a case of squamous cell carcinoma of the esophagus in a 17-year-old male patient who presented with progressive dysphagia and significant weight loss. Contrast-enhanced computed tomography (CECT) neck, chest, and abdomen showed a lesion involving the middle and lower third of the esophagus with contiguous involvement of the gastroesophageal junction (GEJ), extending from D7 to D11 vertebral segments and completely occluding the lumen.

He was managed with chemoradiation, in which three cycles of chemotherapy (Carboplatin 220 mg and Paclitaxel 80 mg) were given, followed by 18 fractions of 40Gy radiotherapy. After this period of chemoradiation, he developed weakness and breathlessness; he was not able to ambulate and became completely bedridden. He underwent a feeding jejunostomy for nutrition, then neoadjuvant chemoradiotherapy (NACRT) and oncological resection with thoracoscopy-assisted transhiatal esophagectomy.

The patient had bubbles in the intercostal tube drain while doing incentive spirometry and basal crepitations, so on the next day, a gastric Conray was done, which showed a suspected contrast leak into bilateral lung fields. CECT neck, chest, and abdomen confirmed the minor leak and was managed conservatively. The abdominal wound developed erythema on the seventh day and was opened showing a small amount of sanguinopurulent discharge, for which daily dressing was done along with antibiotic cefuroxime. On the seventh and eighth days, the patient experienced hoarseness in their voice, a condition managed with supportive care. The patient was successfully discharged after completing all management protocols. The report clearly highlights the need to suspect malignancy in young adults presenting with dysphagia.

## Introduction

Esophageal cancer is one of the common malignancies of the gastrointestinal tract (GIT). As per the World Health Organization (WHO), Globocan 2018, esophageal cancer is the seventh most common cancer (3.2%) in the world and the sixth most common cause of cancer deaths (5.3%). Among the malignancies of the GIT, esophageal cancer accounts for 3.2% of all incident cases of cancer in the world, which is third only after colorectal (10.2%) and stomach (5.7%) cancer. In India, as per WHO, Globocan 2018, esophageal cancer is the sixth most common cancer with an incidence of 5.04%. It is the fifth most common cancer in males and the sixth most common cancer in females, with a male-to-female ratio in India of 2.4:1 [1]. 

Esophageal cancer presents commonly among the elderly with symptoms of dysphagia (74%), weight loss (57%), and odynophagia (17%) [2]. Two main types of cancers have been reported in the esophagus: squamous cell carcinoma (SCC) and adenocarcinoma (AC). Old age has always been noted as a risk factor for esophageal cancer, even after adjusting for gender. The majority of new cases are diagnosed in patients aged 65 to 74 years, with a median age of 67 at the time of diagnosis [3]. The proportion of cases among patients under the age of 45 has been reported to be only 3-10%, while the remaining 90-97% is present in patients above the age of 45 years [4]. This is intuitive because most malignancies result from cell DNA damage accumulating over time and leading to the transformation of proto-oncogenes to oncogenes. Damage can result from biological processes or exposure to risk factors like tobacco smoking and excessive alcohol use [5]. For adenocarcinoma, gastroesophageal reflux disease and obesity are the main risk factors, whereas infection with Helicobacter pylori decreases the risk [5]. Dietary factors may influence the risk of both SCC as well as AC, and the influence of genetic factors is generally low [5]. The current case report is unique as it documents squamous cell cancer of the esophagus in an adolescent from North India who was referred to the general surgery outpatient department (OPD) of Postgraduate Institute of Medical Education and Research (PGIMER), Chandigarh, from a local hospital.

## Case presentation

A 17-year-old male patient presented with chief complaints of progressive dysphagia (Grade 2 progressing to Grade 6) [6], significant weight loss of 14 kg, loss of appetite, and regurgitation episodes over the course of six months. There was no history of retrosternal pain, abdominal pain, awareness of a lump in the body, aspiration, or corrosive intake. On examination, he was conscious, oriented, and hemodynamically stable, and there were no abnormal findings on the neck, per abdominal and per rectal examination. As far as investigations were concerned, he had undergone contrast-enhanced computed tomography (CECT) abdomen at a local private hospital, which showed evidence of an irregular asymmetric circumferential wall thickening involving distal one-third of the esophagus approximately 3.4 cm proximal to the gastroesophageal junction (GEJ), causing luminal narrowing and upstream dilation of the esophagus. The wall thickening extended for a length of approximately 3.4 cm and was 1.5 cm in maximum thickness. The contact area between the tumor and the aorta was 90 degrees. Triangular fat space was preserved with no evidence of locoregional lymph node involvement (T4aN0M0). Upper GI endoscopy (UGIE)-assisted biopsy of the growth confirmed the presence of moderately differentiated esophagus SCC.

**Figure 1:**
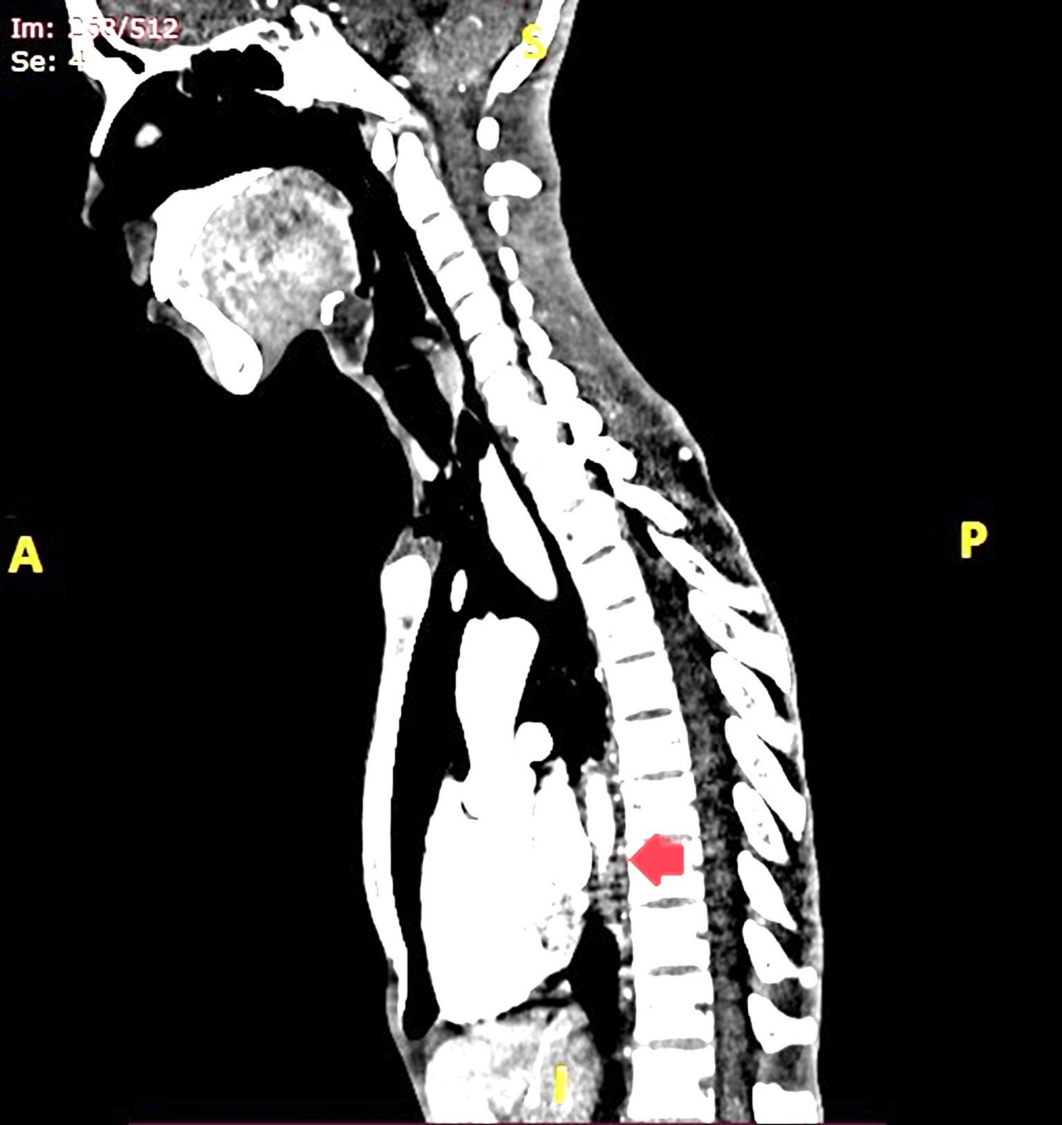
Sagittal section CECT scan of the thorax showing lesion involving the middle and lower third of the esophagus with contiguous involvement of the GEJ, extending from D7 to D11 vertebral segments and completely occluding the lumen (Red Arrow). CECT: contrast-enhanced computed tomography; GEJ: gastroesophageal junction; D7 to D11: dorsal vertebrae 7th to 11th

**Figure 2:**
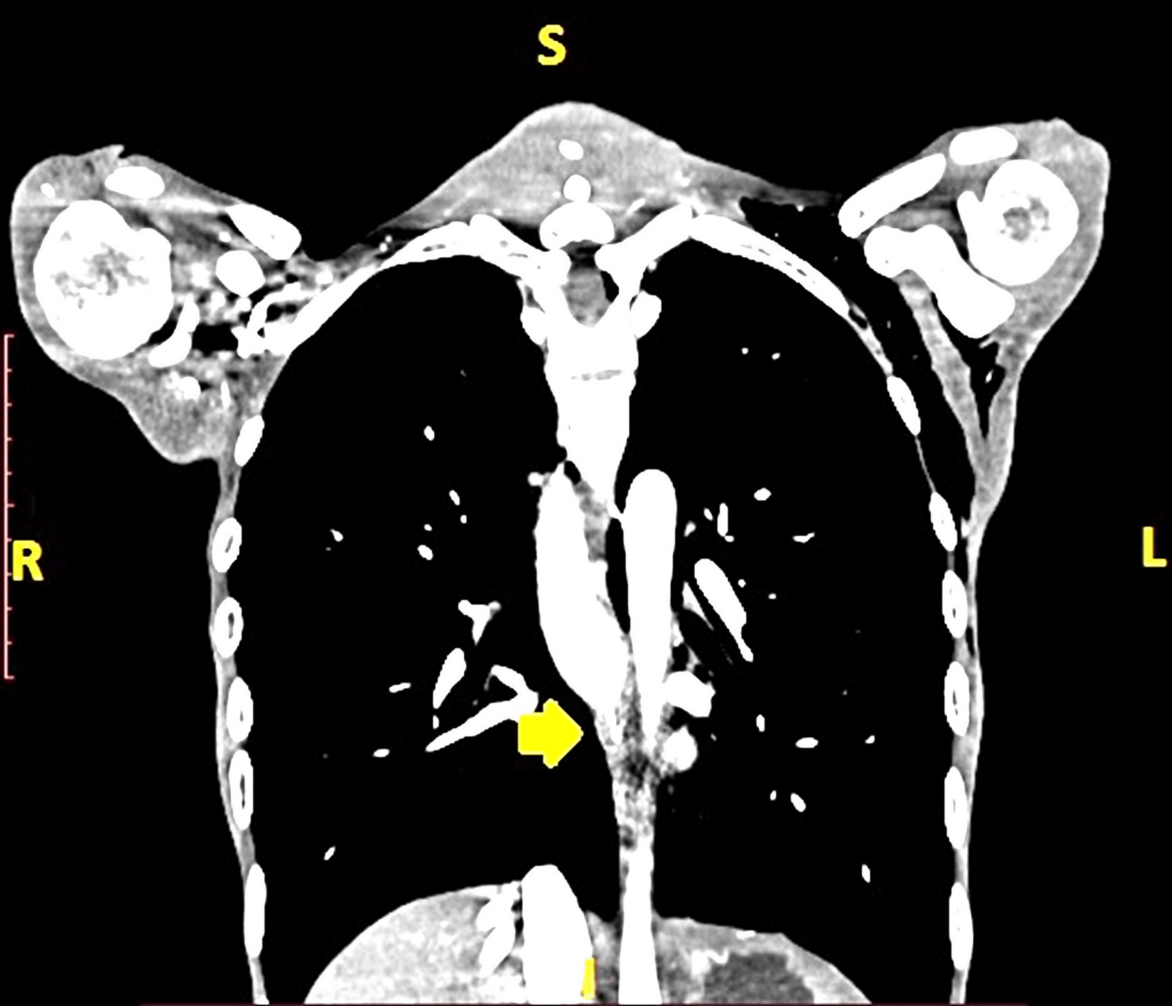
Coronal section CECT scan of the thorax showing lesion involving the middle and lower third of the esophagus with contiguous involvement of the GEJ and completely occluding the lumen (Yellow Arrow). CECT: contrast-enhanced computed tomography; GEJ: gastroesophageal junction

He was then referred to the radiotherapy OPD of the Postgraduate Institute of Medical Education and Research (PGIMER), where pre-neoadjuvant chemoradiotherapy (NACRT) CECT neck, chest, and abdomen were done, followed by three cycles of chemotherapy (Carboplatin 220 mg and Paclitaxel 80 mg). The pre-NACRT CECT showed a lesion involving the middle and lower third of the esophagus with contiguous involvement of the GEJ, extending from D7 to D11 vertebral segments and completely occluding the lumen (Figures 1, 2).

Then, 18 fractions of 40Gy radiotherapy were given, and post-NACRT CECT chest and abdomen was done, which showed a symmetric circumferential mural thickening involving the lower thoracic esophagus at T9 level (thickness 8 mm) and extending inferiorly to involve the GEJ and fundus. The thickening was causing narrowing of the esophagus in this segment with mild dilation of the proximal esophagus. The thickening had a well-defined fat plane with adjacent structures and peri-esophageal nodes not enlarged.

After this period of chemoradiation, he developed weakness and breathlessness; he was not able to ambulate and became completely bedridden. A feeding jejunostomy (FJ) was placed by the modified Whitzel technique through a mini lap, and the patient was admitted for nutritional buildup. Finally, he was planned for thoracoscopy-assisted transhiatal esophagectomy surgery along with cervical, transhiatal, and intercostal tube drain placement. Pre-op CECT neck, chest, and abdomen showed normal cervical esophagus with dilation of the thoracic esophagus in proximal two-thirds with abrupt narrowing of lumen in distal one-third. The narrowing extended from T9 to T10 for a length of 4 cm with mild asymmetrical mural thickening. On thoracoscopy, multiple mediastinal lymph nodes (5x5 cm) and dense adhesions of the lower esophagus with surrounding lungs were seen. In the abdomen, there were no ascites, no omental deposits, the liver and spleen were normal, and GEJ was free of tumors. The cut section of the esophagectomy specimen showed a 4 cm stricturous growth narrowing the whole lumen of the specimen. The lower end of growth was 33 m from GEJ. On histopathological examination, however, the tumor was in GEJ as a thickening of length 44 m with invasion up to the muscularis propria layer, and there was no residual tumor.

The patient was on total parenteral nutrition postoperatively. He was ambulatory, passing flatus and stool and self-voiding on the second postoperative day when the per-urethral catheter was removed and FJ feed was started. The patient had bubbles in the intercostal tube drain while doing incentive spirometry and basal crepitations, so on the next day, a gastric Conray was done, which showed a suspected contrast leak into bilateral lung fields. CECT neck, chest, and abdomen confirmed the minor leak and was managed conservatively. The abdominal wound developed erythema on the seventh day and was opened showing a small amount of sanguinopurulent discharge, for which daily dressing was done along with antibiotic cefuroxime. The patient also had hoarseness of voice on the seventh and eighth days, which was managed with supportive care. Ryle's tube was removed on the eighth day, an oral liquid diet was started on the ninth, and semisolid foods were started on the 13th day. The cervical drain was removed on the fifth day, the transhiatal drain was removed on the 15th day, and the intercostal tube drain was removed on the 21st day, following which the patient was discharged.

## Discussion

The squamous epithelium spans the entire length of the esophagus; hence, SCC can occur anywhere throughout the length and breadth of the esophagus. On the contrary, AC arises from Barrett's metaplasia, which occurs when metaplastic columnar cells replace the stratified squamous epithelium that lines the lower esophagus. About 90% of the esophageal carcinoma among Asians, Africans, and Eastern Europeans is SCC, the remaining 10% being AC [7]. In the Western world, the incidence of SCC has declined steadily over the last thirty years, with a concomitant rise in the incidence of AC [8]. Studies from India show that the proportion of AC varies from 6% to 54% of all esophageal cancers [9].

Overall, esophageal cancer has a male preponderance (7:1) ranging from 11:1 among those aged 50 to 54 to 4:1 among those aged 75 to 79. This predilection for males is seen irrespective of race/ethnicity [9]. A Lancet study comparing the benefit of NACRT plus surgery versus surgery alone in patients with SCC and AC showed a significant increase in five-year survival after a median 45-month follow-up. The current case also underwent this surgery in order to have a maximum survival period postoperatively.

Previous case reports of SCC among adolescents have also been reported in the literature. A 15-year-old boy was diagnosed with SCC and presented with dysphagia of three months duration with a history of betel nut chewing for the previous three years [10]. Lymph nodes were free from tumor cells, and there was no recurrence on follow-up. The youngest patient reported so far was an eight-year-old Indian girl who presented with progressive dysphagia of three months duration [11]. Another case in a 15-year-old female was reported from the Middle East who presented with progressively increasing dysphagia over one month and was treated with esophageal resection. Nine months after surgery, there was no evidence of local recurrence or metastatic disease [12]. Another case in a 14-year-old boy from India was reported treated with definitive irradiation with excellent immediate response [13]. In most of these adolescent cases, no definite etiologic factor could be demonstrated. 

## Conclusions

Factors favoring the development of esophageal cancer are mostly environmental and usually require long periods of exposure. However, cases of esophageal carcinoma in young adults and adolescents brewing in India could indicate the presence of environmental factors or nutritional deficiencies affecting our population. The approach to management is the same as with adults; however, the risk factors might be very different. Esophageal malignancy should be ruled out in any case of dysphagia in Northern India. In addition, further studies on risk factors at a younger age are needed to better understand etiopathogenesis, especially in the Indian context.
